# 基于改性壳聚糖膜净化的超高效液相色谱-四极杆/静电场轨道阱质谱法测定牛奶中5种兽药残留

**DOI:** 10.3724/SP.J.1123.2023.08001

**Published:** 2024-08-08

**Authors:** Wang PAN, Shenping ZHANG, Anqi WANG, Jun HU, Lihui ZHOU

**Affiliations:** 1.华东理工大学化学与分子工程学院, 上海 200237; 1. School of Chemistry and Molecular Engineering, East China University of Science and Technology, Shanghai 200237, China; 2.上海市质量监督检验技术研究院, 国家市场监管重点实验室(乳及乳制品检测与监控技术), 上海 200233; 2. Shanghai Institute of Quality Inspection and Technical Research, Key Laboratory of Milk and Dairy Products Detection and Monitoring Technology for State Market Regulation, Shanghai 200233, China

**Keywords:** 壳聚糖膜, 分散固相萃取, 超高效液相色谱-四极杆/静电场轨道阱质谱, 兽药残留, 牛奶, chitosan membrane, dispersive solid phase extraction (DSPE), ultra-high performance liquid chromatography-quadrupole/electrostatic field orbitrap mass spectrometry (UHPLC-Q/Exactive Orbitrap MS), veterinary drug residues, milk

## Abstract

本研究通过在壳聚糖膜(chitosan membrane, CSM)表面接枝长碳链,获得了十八烷基三甲氧基硅烷改性的壳聚糖膜(C_18_-CSM),将其作为分散固相萃取吸附剂对牛奶基质进行净化,并结合超高效液相色谱-四极杆/静电场轨道阱质谱(UHPLC-Q/Exactive Orbitrap MS)建立了牛奶中氧氟沙星、恩诺沙星、环丙沙星、地西泮和甲硝唑的测定方法。利用扫描电子显微镜、傅里叶变换红外光谱仪和接触角测试仪对C_18_-CSM材料进行表征。对牛奶样品的前处理条件进行优化,最优条件如下:提取溶剂为5%甲酸乙腈,NaCl用量为1 g,萃取次数为1次,C_18_-CSM用量为20 mg。采用Hypersil GOLD VANQUISH色谱柱(100 mm×2.1 mm,1.9 μm)进行分离,以0.1%甲酸水溶液和0.1%甲酸乙腈作为流动相进行梯度洗脱,流速为0.3 mL/min,进样量为1 μL,柱温为25 ℃,在电喷雾电离源正离子模式下进行检测。在优化的前处理条件下,5种兽药在0.5~100 μg/L范围内具有良好的线性关系,相关系数(*r*^2^)≥0.9970,检出限(LOD)为0.2 μg/L,定量限(LOQ)为0.5 μg/L。5种兽药在3个加标水平(0.5、1、5 μg/L)下的回收率为79.5%~115%,日内精密度为7.0%~13%(*n*=6),日间精密度为1.3%~11%(*n*=3)。实验结果表明,本文所建立方法操作简便,准确可靠,能够满足牛奶中5种兽药残留的同时检测。

## Abstract (en)

Milk is an important consumer product with high nutritional value. The presence of veterinary drug residues in milk owing to the indiscriminate use of veterinary drugs may affect consumer health. In the mass spectrometric analysis of trace compounds, chromatographic co-eluting components easily interfere with the mass spectral signals obtained, affecting the accuracy of qualitative and quantitative analyses. Matrix purification is a promising method to reduce the matrix effect. Chitosan is a natural biopolymer with numerous active functional groups such as amino, acetyl, and hydroxyl groups; these groups can adsorb lipids through hydrophobic and electrostatic interactions. Chitosan also has the advantages of low production cost, stable chemical properties, and convenient modification. Novel chitosan-based materials are promising candidates for lipid purification. In this study, a chitosan membrane was modified with trimethoxyoctadecylsilane (C_18_-CSM). C_18_-CSM was prepared through one-step hydrolysis and used as a dispersive solid phase extraction (DSPE) adsorbent to purify the matrix during milk pretreatment. We combined C_18_-CSM with ultra-high performance liquid chromatography-quadrupole/electrostatic field orbitrap mass spectrometry (UHPLC-Q/Exactive Orbitrap MS) to develop an effective method for the extraction and determination of ofloxacin, enrofloxacin, ciprofloxacin, diazepam, and metronidazole in milk. C_18_-CSM was characterized using scanning electron microscopy, Fourier transform infrared spectroscopy, and water contact angle testing. The results indicated that the material has a rough surface and uniformly dense cross-section. The water contact angle of C_18_-CSM was 104°, indicating its good hydrophobicity. The pretreatment conditions (extraction solvent, dosage of NaCl, extraction frequency, and dosage of C_18_-CSM) that influenced the recoveries of the five veterinary drugs were investigated in detail. The optimal conditions were established as follows: 5% formic acid in acetonitrile, 1 g NaCl, extraction 1 time, 20 mg C_18_-CSM. Separation was performed on a Hypersil GOLD VANQUISH column (100 mm×2.1 mm, 1.9 μm). The mobile phase consisted of 0.1% formic acid aqueous solution and 0.1% formic acid in acetonitrile, and was flowed at a rate of 0.3 mL/min. The sample injection volume was 1 μL, and the column temperature was maintained at 25 ℃. Mass spectrometric analysis was performed in positive electrospray ionization mode. To verify the necessity of the purification material, the matrix effect was investigated using the matrix-matched standard curve method. The use of C_18_-CSM reduced the matrix effects of the five necessity drugs from the range of -22%-8.8% to the range of -13%-3.6%, indicating that C_18_-CSM is a highly efficient DSPE material. Under optimal conditions, the developed method showed good linearities within the range of 0.5-100 μg/L, with correlation coefficients (*r*^2^)≥0.9970. The limits of detection(LODs) and quantification (LOQs) were 0.2 μg/L and 0.5 μg/L, respectively. To assess the accuracy and precision of the method, we prepared milk samples with three spiked levels (low, medium, and high). The recoveries of the five veterinary drugs were ranged from 79.5% to 115%, and the intra-day and inter-day relative standard deviations were 7.0%-13% (*n*=6) and 1.3%-11% (*n*=3), respectively. This study provides a simple, accurate, and reliable method for the rapid and simultaneous determination of the five veterinary drug residues in milk.

牛奶含有人体所需的多种营养物质,随着人们生活水平的提高,牛奶已成为我国需求量最大的一种营养品。奶牛在养殖过程中的疾病预防、治疗及圈舍消毒均需使用抗生素或其他兽药,休药期管理不严或挤奶设备污染都容易造成原料乳产生兽药残留,进而通过乳及乳制品等终端产品给消费者健康造成隐患。牛奶中残留的兽药可能会造成过敏反应、诱发细菌耐药性,甚至产生致畸、致癌等危害^[1,2]^。根据《食品安全国家标准 食品中兽药最大残留限量》(GB 31650-2019)^[3]^对禁限用兽药的规定,牛奶中恩诺沙星和环丙沙星的最大残留限量之和为100 μg/kg,甲硝唑和地西泮不得在牛奶中检出。2022年发布的《食品安全国家标准 食品中41种兽药最大残留限量》(GB 31650.1-2022)^[4]^对禁限用兽药进行了增补,规定牛奶中氧氟沙星的最大残留限量为2 μg/kg。由此可见,针对牛奶中兽药残留的检测至关重要。

目前,超高效液相色谱-串联质谱(UHPLC-MS/MS)因具有灵敏度高、测量范围广、结果准确等优点而被广泛应用于兽药残留检测中^[5⇓⇓⇓-9]^。牛奶样品基质复杂,UHPLC-MS/MS极易受到牛奶基质效应的影响,在检测过程中共洗脱基质组分可能会影响目标分析物的电离效率,导致信号受到抑制或增强,从而产生假阳性或假阴性结果,影响方法定性和定量的准确性^[10,11]^。因此,在牛奶样品的前处理过程中,应尽量去除干扰基质,减少其对质谱检测器的污染。常用的前处理方法主要有液液萃取^[12,13]^、固相萃取^[14,15]^和分散固相萃取^[16,17]^,其中分散固相萃取以固相萃取为基础,具备便捷、价格低廉等优点,已在兽药残留分析中得到了广泛应用。常用的分散固相萃取材料包括十八烷基硅烷(C_18_)和乙二胺-*N*-丙基硅烷(PSA)等,其中C_18_主要用于吸附脂肪等含有长碳链的样品基质^[18]^; PSA通常用于去除各种极性有机酸、色素、糖类、脂肪酸和其他一些可以与PSA形成氢键的基质共提取物等,但由于PSA是碱性物质,可能会导致酸性兽药的回收率偏低^[19]^。上述前处理方法主要适用于理化性质相似的某一类兽药,随着对多种不同理化性质兽药残留检测和风险分析需求的增加,现有前处理方法及固相萃取材料已难以满足日渐复杂的基质净化和多种兽药检测要求,因此开发新型净化材料对于不同理化性质兽药的检测具有重要意义。

壳聚糖(chitosan, CS)是一种天然生物高分子聚合物,含有丰富的氨基、乙酰氨基和羟基等活性官能团,其可以通过疏水相互作用和静电相互作用来吸附脂质^[20,21]^,是一种高效的脂肪吸附剂。同时,壳聚糖具有易获得、易于化学修饰、化学性质稳定等优点,以壳聚糖为基础的新型材料已成为一类极具潜力的分散固相萃取材料^[22]^。有研究^[20]^使用废弃虾壳合成了壳聚糖,并将其作为牛奶中多种兽药的分散固相萃取剂;牛奶样品经净化后,磺胺类、苯并咪唑类、大环内酯类等兽药的基质效应从-40%~-10%变为-10%~10%,表明壳聚糖能够有效降低牛奶样品的基质效应。Jung等^[22]^评估了壳聚糖、十八烷基二氧化硅、PSA和增强型脂质去除材料(EMR)对牛肉基质中多类兽药的净化效果,结果发现,壳聚糖处理组中四环素类、磺胺类、喹诺酮类、大环内酯类兽药均获得了良好的回收率。

本研究以十八烷基三甲氧基硅烷改性的壳聚糖膜(trimethoxyoctadecylsilane modified chitosan membrane, C_18_-CSM)作为分散固相萃取材料,对牛奶样品基质进行净化,并结合超高效液相色谱-四极杆/静电场轨道阱质谱(UHPLC-Q/Exactive Orbitrap MS)建立了牛奶中5种兽药(氧氟沙星、恩诺沙星、环丙沙星、地西泮和甲硝唑)残留的检测方法。该方法灵敏且准确度高,为复杂基质中兽药残留的分析检测提供了思路。

## 1 实验部分

### 1.1 仪器、试剂与材料

Thermo Q Exactive PLUS超高效液相色谱-质谱联用系统(包含VANQUISH VF-P10-A液相色谱系统及Orbitrap高分辨质谱)、NEXUS-470傅里叶变换红外光谱分析仪、Helios G4 UC型扫描电子显微镜(美国Thermo Fisher公司);90-1型恒温磁力搅拌器(上海泸西分析仪器有限公司);TGL-16C高速台式离心机(上海安亭科学仪器厂);Sl-0246涡旋振荡器(美国Scientific Industries公司);KS PBC B2氮气浓缩仪(上海冠森生物科技有限公司);KSJ系列真空干燥箱(上海精宏试验设备有限公司);DZF-6050电热恒温鼓风干燥箱(上海一恒科学仪器有限公司)。

壳聚糖(分析纯,中黏度:200~400 mPa·s,上海博飞美科化学科技有限公司);乙酸(分析纯,上海凌峰化学试剂有限公司);正硅酸乙酯(分析纯,上海阿拉丁生化科技股份有限公司);NaOH、十八烷基三甲氧基硅烷(分析纯,上海麦克林生化科技有限公司);无水乙醇、氨水、无水MgSO_4_、NaCl(分析纯,上海泰坦科技股份有限公司);甲酸(色谱纯,国药集团化学试剂有限公司);乙腈(色谱纯,上海星可高纯溶剂有限公司);氧氟沙星、恩诺沙星、环丙沙星、地西泮和甲硝唑标准溶液(100 μg/mL,天津阿尔塔科技有限公司); C_18_(天津博纳艾杰尔科技有限公司)。

### 1.2 CSM和C_18_-CSM的合成

#### 1.2.1 CSM的合成

参考文献[23]报道方法合成CSM。首先将0.3 g壳聚糖粉末溶于10 mL 3%乙酸水溶液中,在室温下进行搅拌,得到均相、透明的壳聚糖溶液,静置消泡,再将溶液倒入玻璃培养皿中,于80 ℃鼓风干燥箱中干燥;待溶液全部蒸发后将培养皿取出,将得到的膜从玻璃培养皿中揭下,并置于0.5 mol/L氢氧化钠水溶液中浸泡0.5 h,再用超纯水洗至中性,置于30 ℃真空干燥箱中干燥,完全干燥后即得到CSM。

#### 1.2.2 C_18_-CSM的合成

参考文献[24]中的方法,在碱性条件下通过一步水解法制备C_18_-CSM。取0.2 g CSM置于含有30 mL乙醇、1.75 mL正硅酸乙酯、1.75 mL十八烷基三甲氧基硅烷的混合溶液中,之后将预先混匀的氨水(3.4 mL)和超纯水(15 mL)快速加入上述溶液中,在室温下搅拌4 h;将膜从溶液中取出,用乙醇洗涤,以除去表面未接枝的十八烷基三甲氧基硅烷,之后在30 ℃真空干燥箱中完全干燥即得到C_18_-CSM;用标准切纸刀将C_18_-CSM裁剪成5 mm×5 mm的正方形膜,待用。

### 1.3 样品前处理

量取5 mL牛奶,置于50 mL离心管中,加入10 mL 5%甲酸乙腈,涡旋30 s,使之充分混合,之后加入1 g NaCl和1 g无水MgSO_4_,涡旋30 s,在5000 r/min下离心5 min;准确量取2 mL上清液于含有20 mg C_18_-CSM的离心管中,涡旋1 min,取1.8 mL上清液,并在50 ℃下用氮气将其溶剂吹干,再加入180 μL乙腈复溶,在9600 r/min下离心5 min,将上清液转移至进样瓶中,进行UHPLC-Q/Exactive Orbitrap MS分析。

### 1.4 基质匹配混合标准溶液的配制

分别移取适量100 μg/mL氧氟沙星、恩诺沙星、环丙沙星、地西泮和甲硝唑标准溶液,用甲醇进行逐级稀释,配制成质量浓度分别为1 mg/L和100 μg/L的混合标准中间液;按照1.3节步骤处理空白牛奶样品,用空白牛奶样品提取液将1 mg/L和100 μg/L的混合标准中间液稀释成系列质量浓度(0.5、1、5、10、20、50、100 μg/L)的基质匹配混合标准溶液。

### 1.5 基质效应的计算

使用基质匹配混合标准溶液和纯溶剂混合标准溶液来评价基质效应(ME),其中纯溶剂混合标准溶液使用50%乙腈水来配制,按照ME=(*S*_m_/*S*_s_-1)×100%来计算基质效应;其中,*S*_m_为基质匹配混合标准曲线的斜率,*S*_s_为纯溶剂混合标准曲线的斜率。若-20%≤ME≤20%,表示存在弱基质效应;若ME<-20%或>20%,表示存在强基质效应。

### 1.6 仪器条件

#### 1.6.1 色谱条件

色谱柱:Hypersil GOLD VANQUISH色谱柱(100 mm×2.1 mm,1.9 μm);柱温:25 ℃;流速:0.3 mL/min;流动相:A相为0.1%甲酸水溶液,B相为0.1%甲酸乙腈。梯度洗脱程序:0~1 min, 5%B; 1~15 min, 5%B~95%B; 15~17 min, 95%B~5%B。进样量:1 μL。

#### 1.6.2 质谱条件

离子源:电喷雾电离(ESI)源,正离子扫描模式;喷雾电压:3200 V;离子源温度:325 ℃;鞘气(N_2_)流速:40 arb;辅助气(N_2_)流速:10 arb;辅助气温度:300 ℃。其他质谱分析参数见表1。

**表1:** 5种兽药的保留时间和质谱参数

Compound	Retention time/min	Quantitative ion (m/z)	Qualitative ions (m/z)
Metronidazole	2.43	285.07892	222.11470, 193.08830, 154.04150
Ofloxacin	4.22	362.15106	318.16123, 261.10338
Ciprofloxacin	4.33	360.17170	316.18197, 245.10847
Enrofloxacin	4.61	332.14050	288.15067, 245.10847, 231.05643
Diazepam	8.97	172.07167	128.04545

## 2 结果与讨论

### 2.1 C_18_-CSM的表征

CSM和C_18_-CSM的红外光谱图如图1所示,3350 cm^-1^处的宽吸收峰归属于O-H和N-H键的伸缩振动,表明CSM和C_18_-CSM中均含有大量的氨基和羟基;1600 cm^-1^处为壳聚糖乙酰基中C=O的伸缩振动峰。对比二者的红外光谱图可以看出,C_18_-CSM在1050 cm^-1^处产生了Si-O键的特征吸收峰,且其在2918 cm^-1^和2878 cm^-1^处的C-H键伸缩振动峰明显增强,以上结果证明了C_18_-CSM的成功制备。

**图1:**
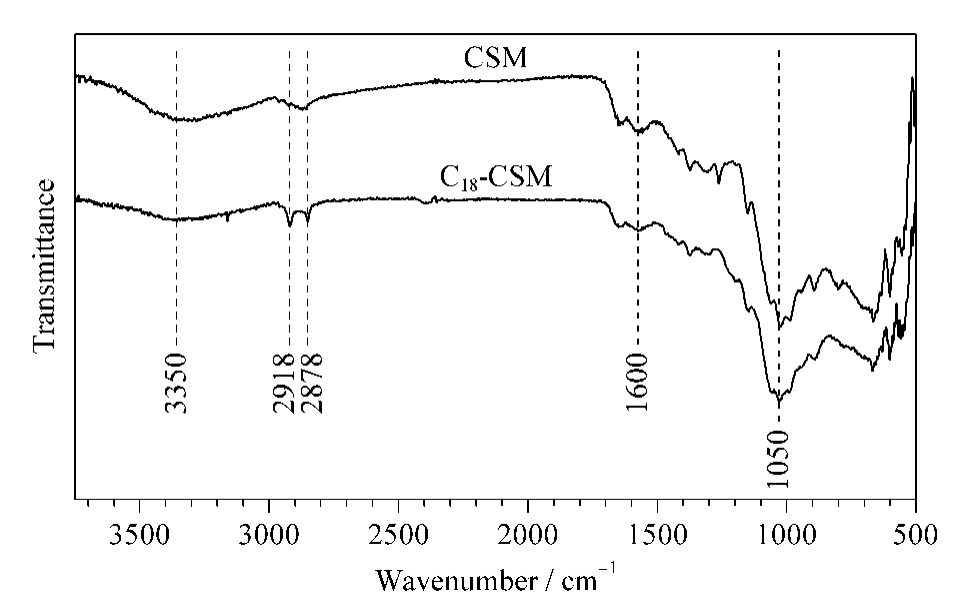
CSM和C_18_-CSM的傅里叶变换红外光谱图

采用扫描电子显微镜对CSM和C_18_-CSM的微观形貌进行观察。从二者的扫描电镜图(图2a和2b)中可以观察到,CSM的表面形貌均匀、光滑、平整,没有孔隙,而C_18_-CSM的表面形貌粗糙。图2c为C_18_-CSM的横截面扫描电镜图,可观察到其横截面是均匀、致密的。

**图2:**
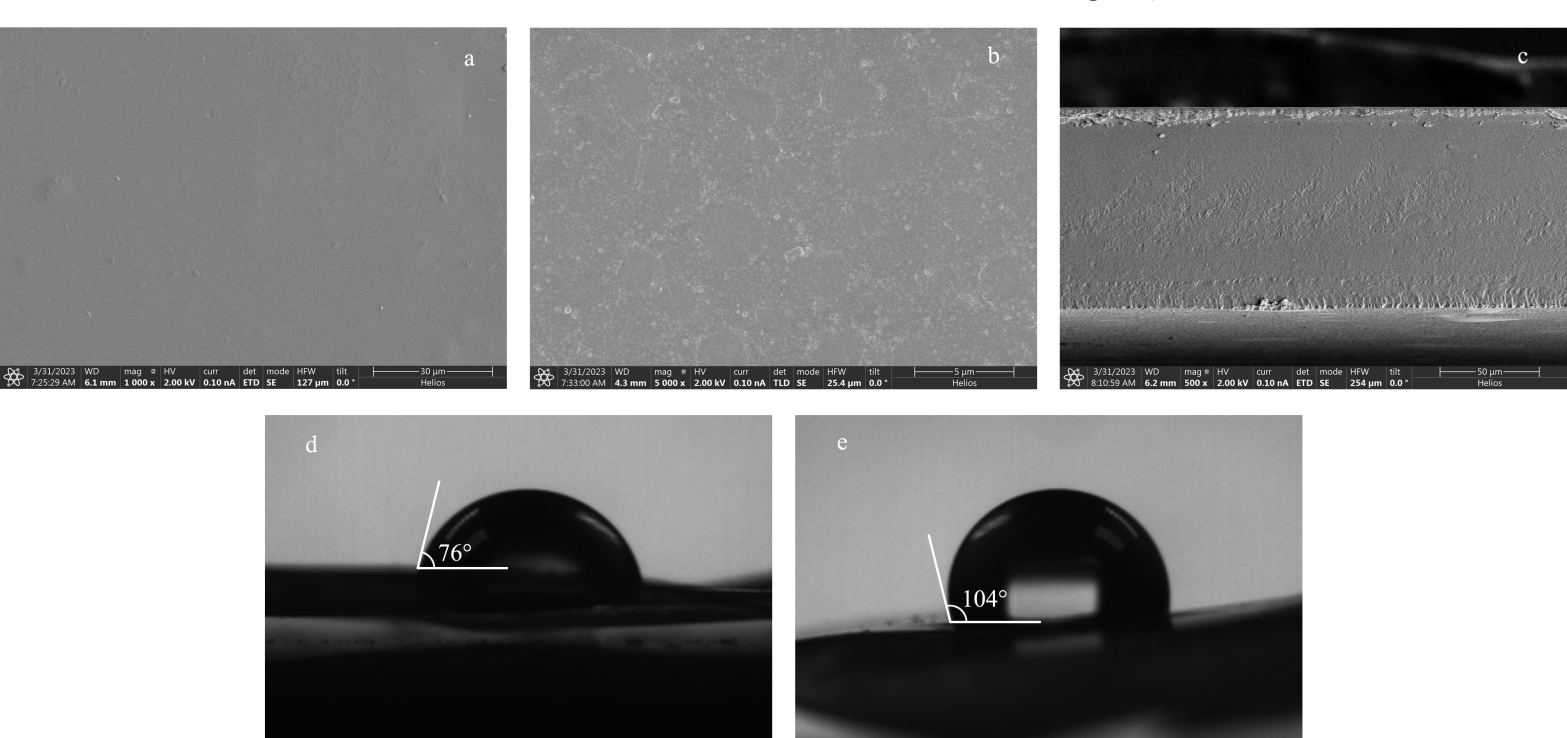
(a)CSM表面、(b)C_18_-CSM表面和(c)C_18_-CSM横截面的扫描电镜图及(d)CSM和(e)C_18_-CSM的水接触角图

CSM和C_18_-CSM的水接触角测试结果如图2d和2e所示,经十八烷基三甲氧基硅烷修饰后,C_18_-CSM的水接触角可达104°,表明其表面成功接枝了长链烷烃,具有一定的疏水性。

### 2.2 样品前处理条件的优化

#### 2.2.1 提取溶剂

乙腈是最常用的提取溶剂,将乙腈与适量的甲酸混合,可以提高两性兽药的回收率;与乙腈相比,经酸化处理后的乙腈溶液还可以有效降低样品提取溶液中的蛋白质含量^[25,26]^。实验考察了不同体积分数(0、1%、3%、5%、8%、10%)的甲酸乙腈对5种兽药提取回收率的影响。从图3a中可以看出,随着甲酸体积分数的增加(0~5%),氧氟沙星、恩诺沙星、环丙沙星和甲硝唑的回收率逐渐增加;当甲酸的体积分数为5%时,5种兽药均获得了良好的回收率;继续增加甲酸的体积分数,5种兽药的回收率均有所下降,因此最终选用5%甲酸乙腈作为提取溶剂。

**图3:**
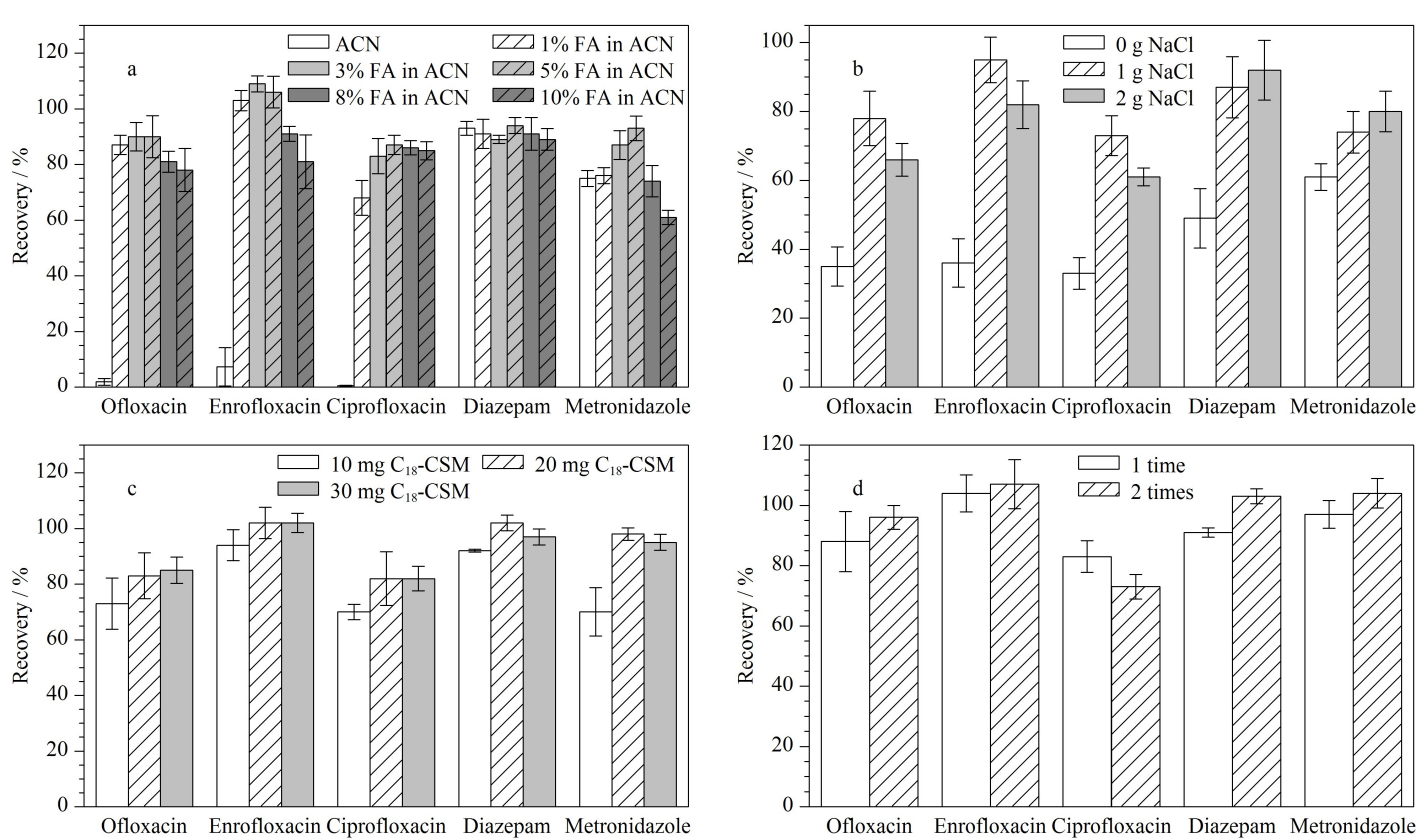
(a)不同体积分数的甲酸乙腈、(b) NaCl用量、(c) C_18_-CSM用量、(d)萃取次数对5种兽药回收率的影响(*n*=3)

#### 2.2.2 NaCl用量

在前处理步骤中加入NaCl可以降低兽药在水相中的溶解度,使更多的兽药转移至有机相中,从而使回收率增大^[27,28]^。实验考察了不同NaCl用量(0、1、2 g)对5种兽药回收率的影响。从图3b中可以看出,不添加NaCl时,5种兽药的回收率较低;当NaCl用量为1 g时,5种兽药的回收率均明显提高;当NaCl用量为2 g时,氧氟沙星、恩诺沙星和环丙沙星的回收率反而降低,这可能是由于NaCl在促进兽药转移至有机相的同时,也能够促进基质成分的转移,当过量使用NaCl时会导致基质效应增大,兽药的回收率降低^[17]^。因此最终确定NaCl的用量为1 g。

#### 2.2.3 C_18_-CSM用量

为了降低基质效应并获得良好的回收率,实验考察了不同C_18_-CSM用量(10、20、30 mg)对5种兽药回收率的影响。如图3c所示,当C_18_-CSM的用量为20 mg时,5种兽药均获得了良好的回收率;继续增加C_18_-CSM的用量,地西泮和甲硝唑的回收率明显降低,因此最终选用20 mg C_18_-CSM用于牛奶样品的前处理。

#### 2.2.4 萃取次数

实验考察了萃取次数(1、2次)对5种兽药回收率的影响。结果如图3d所示,经两次萃取后,除环丙沙星外,氧氟沙星、恩诺沙星、地西泮和甲硝唑的回收率均有所增加,但恩诺沙星、地西泮和甲硝唑的回收率大于100%,可能是由于两次萃取操作会同时增加基质组分的萃取量。因此,为了兼顾5种兽药的回收率及样品前处理效率,选择萃取次数为1次。

### 2.3 吸附剂性能对比

商业分散固相萃取吸附剂C_18_是样品前处理过程中的常用吸附剂。在相同实验条件下,对C_18_、CSM和C_18_-CSM的萃取效果进行比较,结果如图4所示。将C_18_-CSM作为分散固相萃取吸附剂时,5种兽药的萃取效果最好。此外,使用C_18_-CSM萃取5种兽药时,无需利用离心操作,分离过程简便、快捷。因此,本研究合成的C_18_-CSM能够满足牛奶样品的前处理要求。

**图4:**
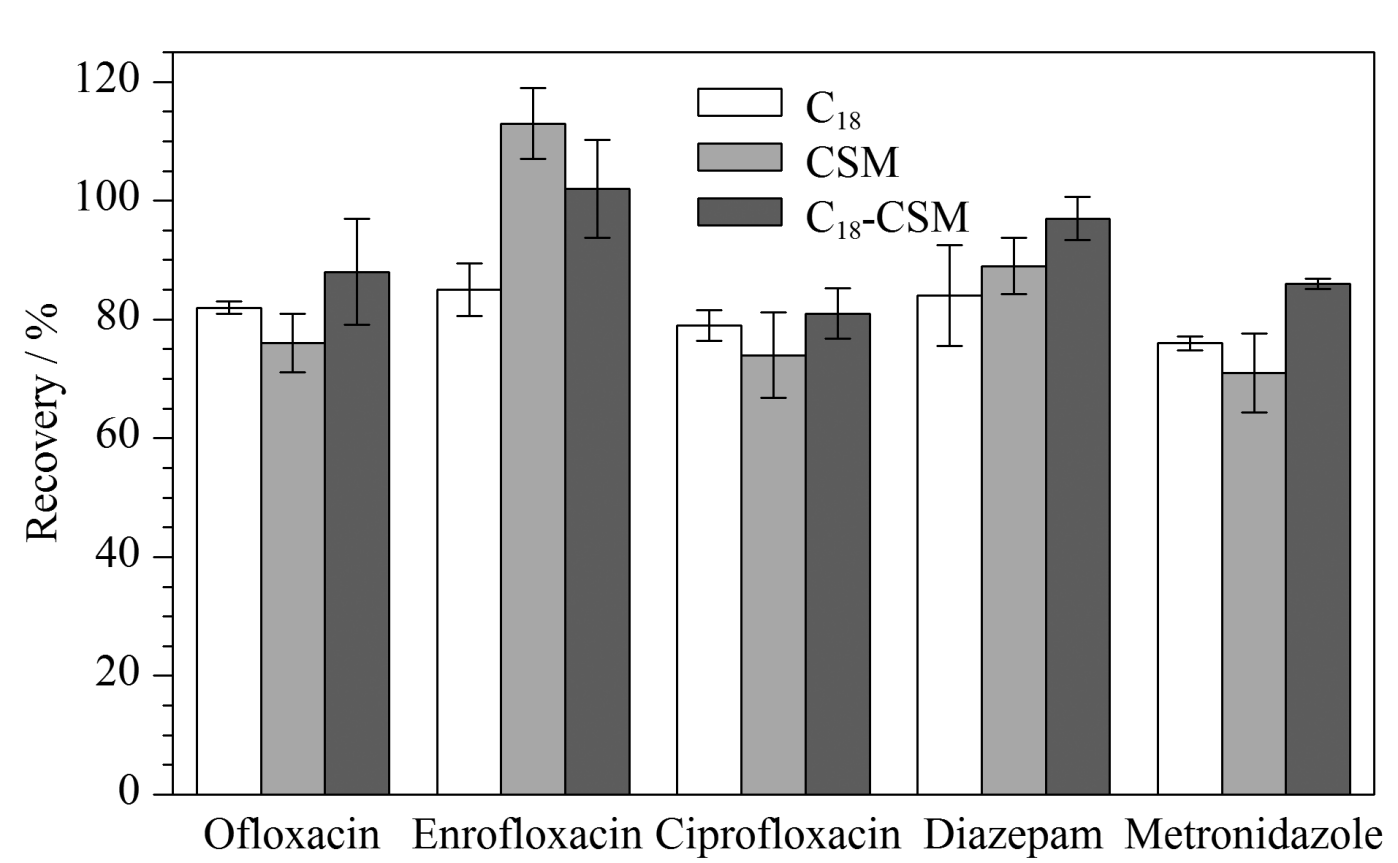
不同吸附剂材料对5种兽药回收率的影响(*n*=3)

### 2.4 方法学考察

#### 2.4.1 线性范围、检出限与定量限

在定性实验中,若目标兽药与相应标准品色谱峰的保留时间相对误差在±2.5%以内,并且二者碎片离子的质量数相对误差在±5×10^-6^以内,则可认为该兽药被检出^[29]^。在空白牛奶样品中加入适量100 μg/L混合标准中间液,逐级稀释后进行测定。以信噪比(*S/N*)≥3且符合上述定性要求的最低质量浓度为检出限(LOD);以*S/N*≥10且满足《实验室质量控制规范 食品理化检测》(GB/T 27404-2008)^[30]^中精密度和准确度要求的最低质量浓度为定量限(LOQ)。结果如表2所示,5种兽药在0.5~100 μg/L内线性关系良好,相关系数(*r*^2^)均≥0.9970, LOD和LOQ分别为0.2 μg/L和0.5 μg/L。

**表2:** 5种兽药的线性方程、线性范围、相关系数、检出限和定量限

Compound	Linear equation	r^2^	LOD/ (μg/L)	LOQ/ (μg/L)
Ofloxacin	Y=1.873×10^5^X+7.583×10^5^	0.9970	0.2	0.5
Enrofloxacin	Y=3.145×10^5^X+6.146×10^5^	0.9987	0.2	0.5
Ciprofloxacin	Y=1.912×10^5^X+3.693×10^5^	0.9986	0.2	0.5
Diazepam	Y=4.063×10^5^X+8.914×10^5^	0.9993	0.2	0.5
Metronidazole	Y=1.906×10^5^X+3.174×10^5^	0.9990	0.2	0.5

*Y*: peak area; *X*: mass concentration, μg/L. The linear ranges of the five veterinary drugs were 0.5-100 μg/L.

#### 2.4.2 基质效应

当使用ESI作为电离源时,牛奶中的基质组分会与目标分析物共同被洗脱,这可能会影响目标分析物的电离效率,造成信号响应的抑制或增强,从而影响方法定量的准确性^[31]^。本文考察了C_18_-CSM对5种兽药基质效应的影响,结果如图5所示。经C_18_-CSM净化后,5种兽药的基质效应从-22%~8.8%变为-13%~3.6%,结果表明C_18_-CSM能够有效降低5种兽药的基质效应。

**图5:**
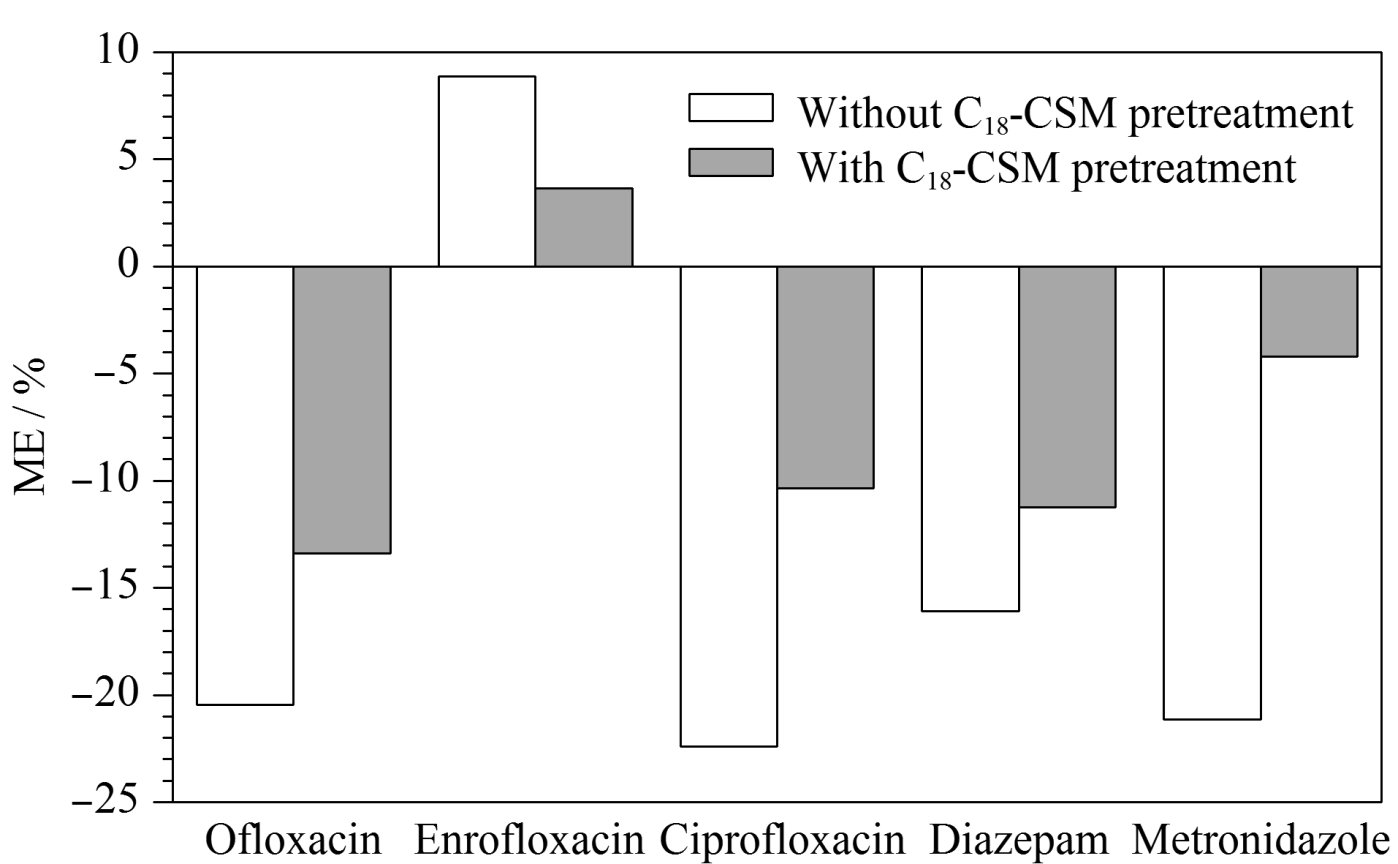
牛奶样品中5种兽药的基质效应

#### 2.4.3 回收率与精密度

在空白牛奶样品中添加不同体积的100 μg/L混合标准中间液,制备成1LOQ、2LOQ和10LOQ 3个加标水平的牛奶样品,进行加标回收试验,每个加标水平平行测定6次,回收率结果见表3。计算相对标准偏差,得到日内精密度(intra-day RSD);连续测定3天,计算日间精密度(inter-day RSD),结果如表3所示。5种兽药在3个加标水平下的回收率为79.5%~115%,日内精密度为7.0%~13%,日间精密度为1.3%~11%。实验结果表明,本方法的准确度和精密度满足GB/T 27404-2008^[30]^要求,可用于牛奶中5种兽药残留的检测分析。

**表3:** 5种兽药的回收率和日内、日间精密度

Compound	Spiked level/ (μg/L)	Recovery/ % (n=6)	Intra-day RSD/% (n=6)	Inter-day RSD/% (n=3)
Ofloxacin	0.5	95.0	13	3.4
	1	104	7.1	1.3
	5	112	9.5	3.2
Enrofloxacin	0.5	93.1	8.5	11
	1	115	13	8.8
	5	115	11	7.3
Ciprofloxacin	0.5	105	12	7.9
	1	90.6	7.0	8.3
	5	103	11	8.7
Diazepam	0.5	79.8	9.8	9.8
	1	91.0	9.5	5.8
	5	83.2	9.0	9.0
Metronidazole	0.5	97.8	9.2	4.3
	1	79.5	7.0	1.4
	5	81.0	10	4.2

### 2.5 与其他方法的比较

与其他文献报道方法相比,本方法具有相对低的定量限、较宽的线性范围和良好的回收率,结果见表4。本方法能够用于牛奶中氧氟沙星、恩诺沙星、环丙沙星、地西泮和甲硝唑的准确测定,为牛奶样品中兽药的残留分析提供了高效、灵敏的分析方法。

**表4:** 本方法与文献报道的牛奶中兽药检测方法的比较

Adsorption material	Detection method	Compounds	Linear range/ (μg/L)	LOQ/ (μg/L)	Recoveries/%	Ref.
C_18_	UPLC-MS/MS	ofloxacin, enrofloxacin, ciprofloxacin	1-100	1	79.3-119	[1]
HLB	UPLC-MS/MS	ofloxacin, enrofloxacin, ciprofloxacin	1-200	1	83.6-119	[7]
		metronidazole	0.1-10	0.1		
HLB	UPLC-MS/MS	ofloxacin	0.5-10	0.8	66.3-91.5	[32]
		enrofloxacin, ciprofloxacin		0.1		
SPE column^*^	HPLC-MS	metronidazole	2.0-50	0.5	71.1-92.1	[33]
		diazepam		0.2		
C_18_-CSM	UHPLC-Q/Exactive	ofloxacin, enrofloxacin, ciprofloxacin,	0.5-100	0.5	79.5-115	this work
	Orbitrap MS	diazepam, metronidazole				

* cation exchange and polystyrene-divinylbenzene SPE column.

### 2.6 实际样品分析

在超市随机选取20份不同品牌的牛奶样品,采用本方法进行测定,结果表明,所有样品均未检出氧氟沙星、恩诺沙星、环丙沙星、地西泮和甲硝唑残留。

## 3 结论

本研究以C_18_-CSM作为分散固相萃取材料,结合UHPLC-Q/Exactive Orbitrap MS,建立了牛奶中氧氟沙星、恩诺沙星、环丙沙星、地西泮和甲硝唑5种兽药残留的分析方法。C_18_-CSM吸附剂的合成方法简单,成本低,基质去除率高,能够有效降低牛奶样品的基质效应。所建方法操作简单,线性范围宽,定量限低,可满足牛奶中氧氟沙星、恩诺沙星、环丙沙星、地西泮和甲硝唑的分析要求。
